# Fœtal Bones Colored with Madder, Administered to a Sow

**Published:** 1829

**Authors:** 


31. *Fœtal bones coloured with Madder, administered to a sow.*—Of late years, the number of authentic facts relative to the absorption of active substances into the circulation, has increased to such an extent, as to redeem our predecessors of the *humoral* school, from much of the ridicule which was at one time cast upon them. Many years ago, Dr. Mussey, now a professor in Dartmouth college, performed and published in Barton's Med. and Phys. Journal, or some other periodical, a series of experiments, going to prove that madder, in solution, is absorbed through the skin. Very lately, [*Am. Jour.*] he has administered the same substance to sows, in a state of *utero-gestation*, and found, that not only their urine, and the serum of their blood, contained the medicine; but also the *liquor amnii*, and the fœtal bones, even to the teeth.

